# Innovation in sepsis trials: rethinking endpoints, statistics and patient stratification

**DOI:** 10.1186/s40635-026-00911-7

**Published:** 2026-06-01

**Authors:** Sascha David, Marc Leone, Massimo Girardis, Mattia M. Müller, Srdjan Gavrilovic, Roberta Domizi, Elisa Damiani, Ignacio Martin-Loeches, Ricard Ferrer, Christian Bode, Benjamin Chousterman, Lene Russell

**Affiliations:** 1 Institute of Intensive Care Medicine, University Hospital Zurich & University of Zurich, Rämistrasse 100, 8032 Zurich, Switzerland; 2 Department of Nephrology, Medical School Hannover, Hannover, Germany; 3 German Sepsis Society (DSG), Berlin, Germany; 4 Department of Anesthesiology and Intensive Care Medicine, Nord Hospital, Assistance Publique Hôpitaux Universitaires de Marseille, Aix Marseille University, Marseille, France; 5 Department of Anesthesia and ICU, University Hospital of Modena, Modena, Italy; 6 Faculty of Medicine, University of Novi Sad, Novi Sad, Serbia; 7 Institute for Pulmonary Diseases of Vojvodina, Sremska Kamenica, Serbia; 8 Clinic of Anesthesia and Intensive Care, Department of Biomedical Sciences and Public Health, Università Politecnica Delle Marche, Ancona, Italy; 9 Clinic of Anesthesia and Intensive Care, Azienda Ospedaliero Universitaria Delle Marche, Torrette, Italy; 10 Department of Intensive Care Medicine, Multidisciplinary Intensive Care Research Organization (MICRO), St James’s Hospital, Dublin, D08 NHY1 Ireland; 11 School of Medicine, Trinity College Dublin, Dublin, D02 PN40 Ireland; 12 Trinity Centre for Biomedical Engineering, Dublin, Ireland; 13 Intensive Care Department, Vall d’Hebron Hospital Universitari, SODIR Research Group, Vall d’Hebron Institut de Recerca (VHIR), Barcelona, Spain; 14 Department of Medicine, Universitat Autònoma de Barcelona, Vall d’Hebron Barcelona Hospital Campus, Passeig Vall d’Hebron 119-129, 08035 Barcelona, Spain; 15 Department of Anesthesiology and Intensive Care Medicine, University of Bonn, University Hospital Bonn, Bonn, Germany; 16 Université Paris Cité, INSERM U942 MASCOT, 75006 Paris, France; 17 Department of Anesthesia and Critical Care, AP-HP, Hôpital Lariboisière, 75010 Paris, France; 18 Department of Intensive Care, Herlev-Gentofte Hospital, Copenhagen, Denmark; 19 Department of Clinical Medicine, Copenhagen University, Copenhagen, Denmark

**Keywords:** Trials, Endpoints, Immunosep, Andromeda, Hemoadsorption, Precision medicine, Immunotherapy

## Abstract

Despite extensive research, effective causal therapies for sepsis remain elusive, highlighting a persistent translational gap between biological understanding and clinical outcomes. Traditional randomised clinical trials (RCTs) have largely failed, likely due to profound patient heterogeneity, dynamic immune trajectories, and reliance on broad syndromic definitions coupled with mortality-centric endpoints. Such designs risk diluting treatment effects and obscuring biologically meaningful signals. Recent studies, however, suggest that precision medicine approaches may overcome these limitations. Trials such as ANDROMEDA-SHOCK-2, IMMUNOSEP, INSPIRE, and TIGRIS have operationalised patient stratification based on physiological or immunological phenotypes and employed novel endpoints or statistical frameworks. ANDROMEDA-SHOCK-2 used capillary refill time guided haemodynamic resuscitation and a hierarchical win-ratio composite endpoint to personalise treatment and enhance sensitivity to benefit. IMMUNOSEP stratified patients by macrophage activation-like syndrome or immunoparalysis, demonstrating improved organ function with targeted interventions using the SOFA score as a primary endpoint. Similarly, INSPIRE focused on progression to organ dysfunction in high-risk pneumonia patients, while TIGRIS employed Bayesian analysis to enrich patients by endotoxin activity, increasing trial efficiency. These studies illustrate that aligning interventions with biological pathways, incorporating adaptive trial designs, and moving beyond short-term mortality can reveal clinically meaningful benefits obscured in conventional trials. Collectively, they signal a conceptual shift in sepsis research towards precision-guided therapies, adaptive protocols, and biologically informed endpoints, suggesting that the era of universal failure may be giving way to more targeted, effective approaches in critical care.

## Introduction

Despite an extensive body of experimental and clinical research, specific treatments for sepsis are still lacking [1, 2]. This persistent gap between biological insight and clinical success has been a source of frustration within the critical care community and has led some to question whether meaningful therapeutic progress in sepsis is achievable at all. At the same time, recent years have seen the emergence of randomised clinical trials (RCT) that deviate from traditional sepsis research paradigms, suggesting that the narrative of universal failure may be incomplete.

The aim of this viewpoint is not to provide an exhaustive systematic review of all sepsis trials conducted to date, but rather to discuss why so many interventions have failed and whether recent methodological and conceptual shifts may offer a way forward. We focus on the hypothesis that repeated trial failure potentially reflects a mismatch between the biological heterogeneity of sepsis and the use of trial designs that rely on broad syndromic definitions, unselected populations, and unreachable mortality-centric endpoints. Importantly, these challenges are not unique to sepsis but reflect a broader limitation of syndromic conditions in critical care. Similar issues arise across a range of inflammatory ICU conditions, where biologically heterogeneous patient populations are grouped under umbrella diagnoses that may obscure differential treatment responses (e.g. ARDS) [3].

In this viewpoint authored by a group of European experts, we did not aim for completeness but instead sought to illustrate broader trends and conceptual shifts within the field. This work is motivated by two primary considerations. First, to challenge the prevailing notion that sepsis is inherently resistant to therapeutic interventions, and second, to highlight some emerging strategies that may allow future trials to detect clinically meaningful benefits, possibly obscured by conventional designs. By examining recent trials that align interventions with biological phenotypes or physiological severity markers and move beyond crude mortality endpoints, we aim to stimulate discussion on how sepsis research and, ultimately, sepsis care could potentially evolve. For clarity, phenotypes refer to subgroups characterised by dominant mechanistic pathways (e.g. immunosuppression measured by HLA-DR expression) and expected differential responses to targeted interventions, whereas severity markers (e.g. lactate or capillary refill time) primarily quantify the extent of organ dysfunction or illness burden without defining the underlying biology.

### Why do we face such a long history of failed sepsis research?

Despite major advances in critical care and a refined conceptual definition of sepsis as life-threatening organ dysfunction driven by a dysregulated host response to infection, the translation of biological insights into effective causal therapies has remained elusive. While early use of supportive strategies such as antimicrobial therapy, source control, and haemodynamic stabilisation has improved outcomes, interventions aimed at modifying the underlying disease process have repeatedly failed to demonstrate consistent benefit [4] but are still widely used in critical clinical situations [5]. Consequently, the secular decline in sepsis mortality has reached a plateau, and in some regions is even reversing [6].

A central explanation for these failures lies in the profound biological and clinical heterogeneity of sepsis. Patients differ widely with respect to infection source, pathogen burden, comorbidities, genetic background, and, critically, their immune trajectory, which may evolve from early hyperinflammation to prolonged immunosuppression within the same individual [7, 8]. RCT enrolling such heterogeneous populations under a single syndromic label inevitably risks diluting potential treatment effects that may only be present in biologically defined subgroups [9].

In parallel, many sepsis trials have relied on short-term mortality as the primary endpoint, an outcome influenced by numerous factors beyond the intervention, including frailty, baseline organ function, and decisions regarding life-sustaining treatment. This focus on short-term mortality, while clinically intuitive, may be poorly suited to detect biologically meaningful effects of targeted therapies, particularly in moderate-sized trials or early-phase studies. As a result, true signals of benefit may have been obscured rather than disproven [10].

Taken together, decades of unsuccessful sepsis trials may reflect less the absence of effective therapeutic targets than the limitations of trial designs that inadequately account for disease heterogeneity, dynamic immune states, and endpoint selection. This recognition provides the conceptual foundation for precision-based approaches that aim to align interventions, patient selection, and outcomes more closely with underlying pathophysiology.

### Can “precision medicine” work in sepsis trials?

Progress in the development of causal therapies for sepsis will likely require a transition towards precision medicine, with patient stratification into distinct (sub)phenotypes and endotypes and the use of integrative, mechanism-informed trial designs. An additional challenge is that these sepsis subphenotypes are not static but may evolve over time within the same patient. Transitions from hyperinflammatory states to immunosuppression, or shifts in haemodynamic profiles, may necessitate adaptation of therapeutic strategies during the disease course.

Over recent months, several RCTs have operationalised this concept in different ways and reported positive signals. These studies not only provide proof of concept but also suggest a shift in how future sepsis trials should be designed and interpreted.

**ANDROMEDA-SHOCK-2** [11] is a large international multicentre clinical trial, which randomised patients with septic shock to either a personalised haemodynamic resuscitation protocol targeting capillary refill time (CRT) or usual care. Using CRT as a surrogate for tissue perfusion, patients in the intervention group received personalised resuscitation focusing on *their predominant cause of haemodynamic instability,* including hypovolemia, vasoplegia and myocardial dysfunction. This approach prioritises the resolution of tissue hypoperfusion and microcirculatory dysfunction over the mere normalisation of macro-haemodynamic parameters such as mean arterial or central venous pressures. By using the CRT as a rapid, easy, bedside surrogate for peripheral perfusion, integrated with other haemodynamic evaluations (including pulse pressure, fluid responsiveness, cardiac function, and considering previous arterial hypertension), clinicians can implement a dynamic feedback loop to titrate conventional therapies in real-time. This allows identification of specific haemodynamic phenotypes, distinguishing patients who require further volume expansion from those who may benefit from vasopressor or inotropic support. Moving beyond the dissociation between macro- and microhemodynamics, this tailored approach aims to optimise tissue oxygen delivery while also minimising the deleterious effects of fluid overload or excessive vasopressor load.

As discussed later, a novel statistical approach within sepsis research, the so-called *hierarchical win ratio* endpoint ranked by clinical priority rather than a classical mortality endpoint was used.

**IMMUNOSEP** [12], a European multicentre RCT that applied a personalised immunotherapeutic strategy based on the presence of (i) macrophage activation-like syndrome (MALS), defined by ferritin levels > 4,420 ng/mL, or (ii) sepsis-induced immunoparalysis, defined by ferritin ≤ 4420 ng/mL in combination with reduced HLA-DR receptors on CD45^+^/CD14^+^ monocytes (< 5000 receptors per cell). Accordingly, two completely different interventions, IL-1R blockade for MALS and recombinant interferon gamma (IFN-γ) for immunoparalysis, were tested against their respective placebos. The primary endpoint was improved organ function by day 9, assessed by a decrease of 1.4 points in the mean Sequential Organ Failure Assessment (SOFA) score from baseline, which was reached by 35.1% of patients in the precision immunotherapy arm vs. 17.9% of patients in the control arm (difference 17.4 (CI 6.8–27.2) %, *p* = 0.002). The use of change in SOFA score as primary endpoint captures the trajectory of organ recovery, offering a quantitative, continuous measure that improves statistical power in a moderate-sized phase II study. The choice of a *1*.*4-point* difference was driven by prior empirical data and historical trials such as MAXSEP that have shown that differences in mean SOFA scores at day 9 correlate with 28-day mortality, and a reduction of approximately 1.4 points was considered both *clinically meaningful* and statistically detectable with feasible sample sizes [13]. Once again, considering only mortality at day 28 as primary endpoint would have masked the positive signal in this trial. From our point of view, one advantage of choosing a SOFA-based endpoint is the acknowledgement of organ dysfunction as a central biological target in sepsis research.

Along the same lines, **INSPIRE**, a very recent phase IIa RCT, did test IL-1R blockade in a subgroup of 60 hospitalised patients with pneumonia and signs of organ dysfunction (qSOFA) that were included based on plasma presepsin (soluble CD14) levels [14]. These patients were at the transition between infection and sepsis, giving the authors the opportunity to combine a treatment strategy with a preventative note. This was achieved by focusing on a rather unexplored endpoint, i.e. the disease progression to organ dysfunction at day 7 or death at day 90. Despite the small explorative setting, there was a significant difference of 30.0% [5.9–49%] in favour of the treatment over placebo. Even the 90-day mortality was 43.3% and 20% (difference 23.3% [0–43.7%]; *p* 0.029).

Last, the **TIGRIS** trial is a North American multicentre RCT investigating polymyxin B (PMX)-based endotoxin adsorption in patients with septic shock stratified by elevated endotoxin activity (EA) [15]. TIGRIS specifically enrolled patients with endotoxic septic shock identified by an intermediate range of endotoxin activity (EA: 0.60–0.89). This enrichment strategy was based on post hoc subgroup analyses [16] from the earlier EUPHRATES trial [17] identifying that patients within this range were more likely to benefit from PMX hemoadsorption. Rather than using a standard frequentist design, TIGRIS employs a Bayesian analytical approach. This meant the trial incorporated patients with EA 0.60–0.89 from the previous RCT as a prior distribution in its statistical model. By formally combining historical evidence about treatment effects with new data, the Bayesian design increases power and efficiency, potentially allowing it to reach meaningful conclusions with a smaller sample size and providing a posterior probability of benefit rather than relying solely on traditional p‑values, which reduce inference to a binary cut-off between “significant” and “non-significant”. At 28 days, 39% of patients in the PMX group and 45% of patients in the control group had died, yielding a posterior probability of benefit of 95·3%. At 90 days, the posterior probability of benefit was even higher, i.e. 99.4% (0.54 [0.32–0.87]).

### The dawn of novel endpoints and statistical approaches in sepsis trials

The three studies were notable not only for their personalised approach, but also for their use of endpoints and statistical methodologies that differ from classical trials. For example, ANDROMEDA-SHOCK-2 used a hierarchical, clustered composite endpoint for which a *win ratio* was calculated. This approach, originally developed in the field of cardiology, was conceived to mitigate a key limitation of classical composite endpoints, namely their focus on each patient’s first event while failing to account for subsequent, potentially more serious or fatal events [18]. To avoid this so-called “time-to-first event” bias, the authors used a hierarchical composite structure, with mortality at the highest level, followed by duration of vital support and length of hospital stay. Each patient is compared pairwise with every patient in the opposite group, starting with mortality. In the event of a tie, the comparison proceeds to the next hierarchical levels, where patients are compared according to duration of vital support and, subsequently, length of hospital stay, with shorter durations considered more favourable. This allows treatment effects across multiple outcomes to be investigated and may enhance statistical power.

However, although the concept of winning or losing for a treatment versus control group is intuitive, precise estimation of effect size and its translation into clinical relevance remain challenging [19]. As a statistically positive result does not necessarily equate to a clinically meaningful benefit, such findings are best interpreted alongside classical single-endpoint analyses, which provide clearer measures of effect magnitude, such as hazard or odds ratios. Indeed, the authors of ANDROMEDA-SHOCK-2 demonstrated in their secondary analyses of individual outcomes that neither mortality nor length of hospital stay showed a meaningful treatment effect, whereas vital support-free days exhibited the most noteworthy benefit associated with the intervention.

Innovative statistical approaches, such as win ratio-based analyses, may increase sensitivity for detecting treatment effects in heterogeneous populations, but they can be limited in their ability to convey the absolute magnitude and clinical relevance of those effects. While this brief discussion does not encompass the full spectrum of emerging endpoint and analytical strategies used in recent trials, the win-ratio framework illustrates a broader shift in contemporary sepsis research. As trial designs and statistical methods become more sophisticated, interpretation must extend beyond a binary view of statistical significance towards a more nuanced appraisal of effect size, uncertainty, and clinical importance. This perspective will be essential for translating methodological innovation into meaningful improvements in the care of patients with sepsis.

### How to implement these findings?

The findings discussed in these trials imply that there may be benefits in moving beyond rigid, one-size-fits-all guideline implementation towards more personalised treatment strategies that acknowledge patient heterogeneity. At the same time, it is neither realistic nor desirable to abandon protocols altogether, given the need for rapid decision-making and consistent, high-quality care in the ICU. Rather, the challenge is to develop adaptive, patient-centred protocols that incorporate predefined decision points and physiological or biological markers within a structured framework. In this way, personalisation and protocolisation should be viewed not as competing paradigms, but as complementary elements of a modern approach to sepsis care that enables both flexibility and reliability at the bedside.

Next, there is a need to reconsider the usefulness of multifactorial and unspecific endpoints such as 28-day or 90-day mortality as the primary goal for the majority of RCTs. Mortality in sepsis often results from a complex interplay of factors beyond the intervention under study, which cannot be fully stratified for, including comorbidities and frailty [20]. Furthermore, survival alone fails to reflect outcomes important to patients, such as long-term functional status, cognitive impairment, quality of life, or disability, which are increasingly recognised as critical endpoints in sepsis research [21]. As a result, mortality is a blunt endpoint that may not accurately reflect the biological effect of a targeted therapy. The focus on organ dysfunction scores or hierarchical win ratios can, under the right conditions, serve as a surrogate for defining the success of an intervention and provide a pathway for stepwise improvement of clinical practice. Even if less meaningful, these endpoints can provide a direction for future research, and one may hypothesise that by continuing to target predefined subpopulations with personalised interventions, we might also be able to change mortality rates in the next few years.

Despite these promising developments, several important challenges remain. First, sepsis is a time-sensitive condition, and variability in assessment of disease onset in clinical trials may obscure treatment effects. Second, sepsis subphenotypes and endotypes are dynamic and may evolve over the course of illness, suggesting that static stratification at enrolment may be insufficient and that adaptive therapeutic strategies could be required during the disease course. Third, the success of targeted interventions depends on prior optimisation of dose, duration, and route of administration, particularly considering the compartmentalised nature of the host immune response. In addition, the feasibility and cost-effectiveness of precision-based approaches must be considered, especially in low- and middle-income settings where the burden of sepsis is high. Together, these considerations highlight key areas for future research and refinement of trial design.

## Summary and conclusion

We argue that decades of non-conclusive sepsis trials reflect not only profound biological and clinical heterogeneity and evolving immune states, but also conceptual frameworks that have prioritised syndromic classification over biological actionability. While the current sepsis definition formally reframes sepsis as organ dysfunction resulting from a dysregulated host response, this construct has proven insufficiently informative at the bedside and unhelpful for therapeutic stratification, effectively collapsing diverse biological trajectories into a single clinical label. The downstream consequence has been trial designs anchored to broadly defined populations and multifactorial mortality endpoints, in which meaningful treatment effects are systematically diluted.

In contrast, the trials discussed here illustrate novel approaches for moving beyond syndromic definitions towards precision medicine approaches that enrich patient selection, align interventions with dominant pathophysiology, and adopt alternative endpoints such as organ dysfunction trajectories or hierarchical composite outcomes. These studies demonstrate that personalised interventions, coupled with innovative statistical methods, can uncover clinically relevant benefits that conventional mortality-driven designs fail to detect. Although this discussion has focused on sepsis, the concepts outlined here likely extend to a broader spectrum of inflammatory and critical illness syndromes.

Taken together, they signal a methodological and conceptual shift in sepsis research, from defining sepsis to deconstructing it, and from uniform treatment strategies to stratified care, adaptive protocols, and biologically meaningful definitions of therapeutic success.

## Data Availability

Not applicable.
